# Zinc Finger Protein CTCF Regulates Extracellular Matrix (ECM)-Related Gene Expression Associated With the Wnt Signaling Pathway in Gastric Cancer

**DOI:** 10.3389/fonc.2020.625633

**Published:** 2021-02-16

**Authors:** Chenbin Liu, Linyi Deng, Jinrong Lin, Jianjun Zhang, Shu Huang, Jinglin Zhao, Peipei Jin, Peiqing Xu, Peihua Ni, Dakang Xu, Le Ying, Yiqun Hu

**Affiliations:** ^1^ Faculty of Medical Laboratory Science, Ruijin Hospital, School of Medicine, Shanghai Jiao Tong University, Shanghai, China; ^2^ Department of Sports Medicine, Huashan Hospital, Fudan University, Shanghai, China; ^3^ Centre for Innate Immunity and Infectious Diseases, Hudson Institute of Medical Research, Clayton, Victoria, Hudson Institute of Medical Research, Clayton, VIC, Australia; ^4^ Department of Molecular and Translational Science, Monash University, Clayton, VIC, Australia

**Keywords:** gastric cancer, zinc finger protein CTCF, extracellular matrix (ECM), Wnt signaling, histone modification

## Abstract

Gastric cancer (GC), a leading cause of cancer-related death, is a heterogeneous disease. We aim to describe clinically relevant molecular classifications of GC that incorporate heterogeneity and provide useful clinical information. We combined different gene expression datasets and filtered a 7-gene signature related to the extracellular matrix (ECM), which also exhibited significant prognostic value in GC patients. Interestingly, putative CCCTC-binding factor (CTCF) regulatory elements were identified within the promoters of these ECM-related genes and were confirmed by chromatin immunoprecipitation sequencing (ChIP-Seq). CTCF binding sites also overlapped with histone activation markers, indicating direct regulation. In addition, CTCF was also correlated with the Wnt signaling pathway. A comparison of human GC cell lines with high or low expression of ECM-related genes revealed different levels of tumor aggressiveness, suggesting the cancer development-promoting functions of ECM-related genes. Furthermore, CTCF regulated COL1A1 and COLA31 expression *in vitro*. Silencing CTCF or COL1A1/COL1A3 markedly inhibited cell growth and migration in the metastatic GC cell line BGC823. Collectively, this ECM-related 7-gene signature provides a novel insight for survival prediction among GC patients. The zinc finger protein CTCF regulates ECM-related genes, thereby promoting GC cell growth and migration.

## Introduction

Gastric cancer (GC) is the fourth most common cancer in the world and causes approximately 738,000 deaths each year, making it the third leading cause of cancer-related death worldwide (1–4). East Asia is recognized as a high-risk region probably due to the high incidence of *Helicobacter pylori* (*H. pylori*) infection and cancer-associated dietary habits. The 5-year relative GC survival rate of all Surveillance, Epidemiology, and End Results (SEER) stages combined is approximately 32%. Early detection and reliable screening are of vital importance in improving the survival rate. Although there are more new insights into the molecular basis of GC than ever before, currently available diagnostic and therapeutic methods do not significantly improve the overall survival (OS) of GC patients. The use of disease risk stratification (tumor-node-metastasis (TNM) stage) and histological grade based on tumor size, lymph node or distant metastasis is not sufficient to determine the prognosis of a given GC patient. The discovery and identification of molecular subtypes and novel biomarkers play an important role in clinical decision making for more effective and selective treatment. Molecular biomarkers have potential values as diagnostic and prognostic tools in GC. It is worthwhile to investigate the molecular mechanisms of GC and to identify novel and specific biomarkers and targets.

Significant progress has been made in identifying novel molecular subtypes of GC over the last few years. The mechanism of GC pathogenesis in these subsets remains unclear and relies on multiple factors. Activation of multiple oncogenic signaling pathways, including the Wnt/β-catenin, PI3K/Akt, ERK/MAPK, and JAK/STAT3 pathways, is common in the process of carcinogenesis. Among these abnormal carcinogenic signaling pathways, the Wnt/β-catenin signaling pathway has attracted considerable attention, and its dysregulation plays an important role in the tumorigenesis of GC. The Wnt signaling pathway is a central regulatory pathway that regulates gene expression. The pathway has an essential role in embryonic development but also functions in a variety of cellular processes, including proliferation, differentiation, migration, and stemness. The Wnt family currently contains 19 members and regulates three pathways: the canonical pathway, the planar cell polarity pathway, and the Ca^2+^ pathway (5). Genomic statistics indicate that deregulation of the Wnt/β-catenin pathway is noted in 46% of gastric tumors (6). Studies have demonstrated that WNT6 and Cav1 together react positively in the chemoresistance of cells to DNA-damaging anthracycline drugs through the activation of the canonical Wnt/β-catenin pathway (7). Cancer generally demonstrates rapid and anomalous growth through extracellular matrix (ECM) remodeling by collagen type I α 1 (COL1A1) and its collagen family members, and the ECM is also highly activated by the Wnt pathway (8, 9). Cell surface receptors transduce signals into cells from the ECM, which regulate diverse cellular functions, such as survival, growth, migration, and differentiation, and are vital for maintaining normal homeostasis; dysregulation of the ECM could lead to cancer progression. However, the underlying Wnt-regulated ECM target gene expression programs in GC are unknown. Further, there is a lack of knowledge regarding how Wnt regulates target gene expression *via* transcription factors (TFs) and histone modifications (HMs).

CTCF, a well-known transcription factor that is essential in organizing chromatin into highly self-interacting, topologically associated domains, has recently become of great interest to researchers in the cancer field (10). CTCF overexpression has been identified in breast cancer (11), cervical cancer (12), ovarian cancer (13), and hepatocellular carcinoma (14). Some mutations are related to tumors (15). In particular, hotspot binding site mutations occur at quite a high frequency (25%) in GC, and these mutations are associated with alterations in gene expression (16). However, the expression pattern of CTCF and its target genes and its roles in GC growth and metastasis remain unknown. Thus, work sought to identify and characterize the initiating molecular subtypes for GC tumors. Indeed, a recent chromatin profiling study to identify enhancers revealed molecular subtypes in other cancers, but no comparable dataset currently exists for GC.

In the current study, we systematically integrated RNA sequencing (RNA-Seq), chromatin immunoprecipitation sequencing (ChIP-Seq) and tissue microarray (TMA) data to identify ECM subtype gene signatures regulated by the zinc finger protein CTCF transcription factor, which is also linked to the Wnt signaling pathway. CTCF target genes that have an important impact on tumor phenotype and prognosis were validated using GC cell line experiments. Cell viability and migration assays showed that silencing CTCF, COL1A1, and COL1A3 inhibited the growth and migration of GC tumor cells.

## Materials and Methods

### Gene Expression Profile Data

The microarray dataset GSE79973 was downloaded from the GEO database (Gene Expression Omnibus (GEO), RRID : SCR_005012). This dataset contained 10 paired GC tissues and adjacent nontumor tissues from GC patients who underwent radical gastrectomy for GC at Zhejiang Provincial People’s Hospital from May 2011 to June 2012.

Raw RNA sequencing data were downloaded from the TCGA (The Cancer Genome Atlas, RRID : SCR_003193). A total of 375 GC samples and 32 matched nontumor samples were obtained for further analysis. Clinical parameters, including age, gender, grade, *H. pylori* infection status and TNM stage (8th edition in 2016), were also downloaded.

### Identification of DEGs

For analysis of the validation cohort from the GEO GC dataset, the LIMMA (17) package (LIMMA, RRID: SCR_010943) of the Bioconductor project in R software was applied to screen the differentially expressed genes (DEGs). We set the cut offs as |log2 fold change (FC)| ≥1 and false discovery rate (FDR) < 0.01 for filtering the DEGs. To analyze the validation cohort from the TCGA GC dataset, we used the edgeR (18) Bioconductor project package (edgeR, RRID : SCR_012802). Genes with |log2FC| ≥ 2 and FDR < 0.01 were considered to be significantly differentially expressed. The overlapping DEGs between the GEO dataset and the TCGA dataset were retained for further study.

### Kyoto Encyclopedia of Genes and Genomes (KEGG) Enrichment, Protein-Protein Interaction (PPI) Network and Module Analyses

KEGG analysis was conducted using the Database for Annotation, Visualization and Integrated Discovery (DAVID) (DAVID, RRID : SCR_001881). The PPI network was constructed using a widely used online database, the Search Tool for the Retrieval of Interacting Genes (STRING) (STRING, RRID : SCR_005223). Cytoscape software was used to visualize the PPI networks (19). The molecular complex detection (MCODE) (MCODE, RRID : SCR_015828) plugin with degree cutoff = 2, node score cutoff = 0.2, k-score = 3, and max depth = 100 was utilized for subset clustering (20).

### Survival Analysis of Hub Genes

The Kaplan-Meier method was used for survival analysis and was performed using the Gene Expression Profiling Interactive Analysis (GEPIA) (Gene Expression Profiling Interactive Analysis, RRID : SCR_018294) web server (21).

### Transcription Factor Binding Site Prediction

Profiles of the transcription factor binding sites were retrieved from the JASPER database. CiiiDER was used to predict transcription factor binding sites across regions of interest (22). Overlapping genes were analyzed to identify common regulatory transcription factors with an upstream scan limit=2,000 bases, a downstream scan limit=500 bases, and deficit threshold=0.15.

### Mapping Transcription Factor Binding Sites

We downloaded the call set from the Encyclopedia of DNA Elements (ENCODE) portal (https://www.encodeproject.org/) (Encode, RRID : SCR_015482) with the following identifiers: ENCBS630AAA. UCSC Browser (http://genome.ucsc.edu/) (UCSC Genome Browser, RRID : SCR_005780) was used to visualize the mapping results.

### Single-Sample Gene Set Enrichment Analysis (ssGSEA) of Transcription Factors

CTCF gene expression was input into the GSEA tool (http://www.broadinstitute.org/gsea/index.jsp) (Gene Set Enrichment Analysis, RRID : SCR_003199) to calculate the separate enrichment score.

### Pathology Analysis of Hub Genes

The immunohistochemistry (IHC) staining images of COL3A1, SPP1, and THBS2 from GC tumor and normal gastric tissues were downloaded from the Human Protein Atlas (HPA) (HPA, RRID : SCR_006710) database (http://www.proteinatlas.org/) for further analysis (23–25). QuPath software (QuPath, RRID : SCR_018257) is used for image analysis (26).

### Cell Culture and Transfection with siRNA

The human GC cell lines HGC27, TGBC11TKB, LMSU, NCC-StC-K140, FU97, MKN45, AGS, HGC27, and BGC823 were purchased from the American Type Culture Collection (ATCC; Manassas, VA, USA) (ATCC, RRID : SCR_001672), CellBank Australia, or Sigma-Aldrich and cultured in RPMI 1640 medium (Invitrogen, CA, USA) supplemented with 10% fetal bovine serum (Invitrogen, CA, USA) and incubated at 37°C and 5% CO_2_. CTCF, COL1A1, and COL3A1 were selected from the upregulated gene groups. A commercially available RNA interfering panel targeting the above genes using SMARTpool siRNA, non-target siRNA, and negative control siRNAs were purchased from GenePharma (Shanghai, China). Individual siRNA sequences are presented in **Supplementary Table 1**. siRNA was used at a concentration of 50 pM and transfected into HGC27 and BGC823 cells to account for the influence of transfection itself. Real-time qRT-PCR was performed to detect the expression of the above genes to verify the effectiveness of knockdown.

### Quantitative Real-Time PCR Analysis

Total RNA was extracted using TRIzol reagent (Invitrogen, USA) according to the instructions. The concentration and quality of RNA were determined using a Nanodrop spectrophotometer (Thermo Fisher Scientific, USA). Reverse transcriptional mRNA expression was analyzed using PrimeScript RT Master Mix (TaKaRa, Japan). For RNA expression analysis, total RNA was first transcribed using the Mir-X™ RNA first-strand synthesis kit (TaKaRa, Japan). Real-time PCR SYBR Premix Ex Taq (TaKaRa, Japan) was replicated thrice in a 7900 HT Real-Time PCR system (Applied Biosystems, USA), and GAPDH expression levels were used as an endogenous control. The results were determined using the 2^-ΔΔct^ calculation method. See **Supplementary Table 2** for primer information.

### MTT Assay

The cells were plated in culture plates and incubated in a CO_2_ incubator. Next day, according to the experimental requirement, at respective time points, 50 µl MTT solutions from the stock (5 mg/ml) was added, and cells were incubated in a CO_2_ incubator in the dark for 2 h. The medium was removed, and formazan crystals that formed in the cells were dissolved using 500 µl of DMSO followed by transfer to a 96-well plate. Metabolically active cells reduced the MTT to blue formazan crystals, which were dissolved in DMSO. Absorbances were measured at 560 nm. Cell viability was calculated as a percentage of vehicle control using GraphPad Prism v.5.0.

### Cell Wound Healing Assay

Cells were plated at confluence in 24-well plates. A wound was created by scraping the confluent monolayer with a p200 pipette tip, and cells were grown in medium supplemented with 1% FBS. The migration of the cells was recorded at 0, 24, and 48 h using a MicroFire camera fitted to an Olympus Inverted Phase Microscope (Olympus) at 10X magnification. The area of the uncovered wound gap was measured using ImageJ software, and details are described in our previous studies (27, 28). All experiments were repeated in triplicate.

### Statistical Analysis

All statistical analyses were performed in R software (version 3.6.3) and GraphPad Prism 7 software. All statistical tests with P < 0.05 were statistically significant.

## Results

### Workflow to Identify Key Genes and Pathways in GC

**Figure 1:**
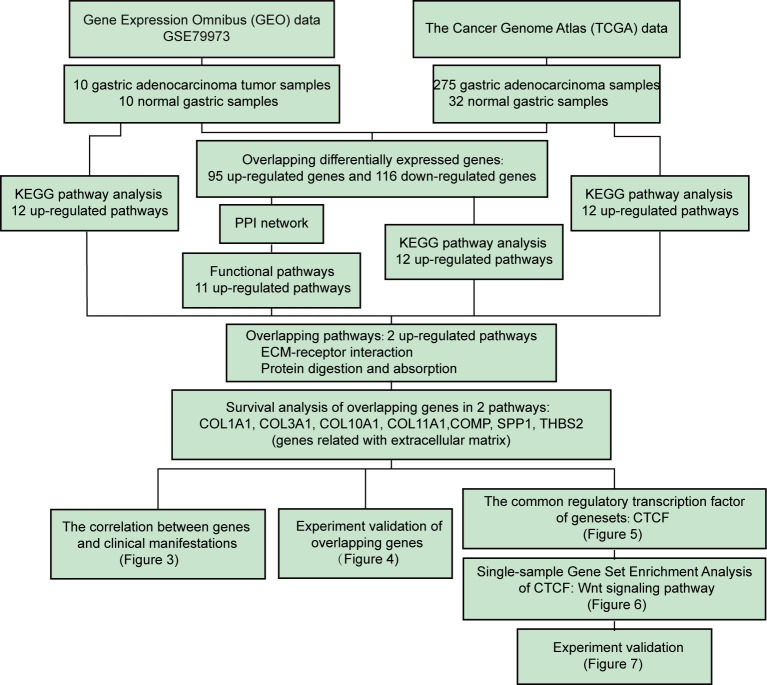
Flow chart of the systematic comprehensive analysis of differentially expressed genes (DEGs) from The Cancer Genome Atlas (TCGA) cohort and the Gene Expression Omnibus (GEO) series of gastric cancer (GC) patients and correlation of DEG expression with overall survival (OS). Further investigation revealed that the extracellular matrix (ECM) signature regulates gastric cancer (GC) progression through the Wnt pathway, and this result was validated by mRNA expression, cell viability and migration assays using a siRNA strategy in aggressive GC cell lines.

We aimed to identify the DEGs between tumor and normal tissues, establish clinically relevant molecular subtypes and identify significant signatures for GC. First, RNA-Seq data from the GEO and TGCA databases were used to identify DEGs. The overlapping DEGs from two databases were used for KEGG pathway and PPI network analyses. The top 12 up-regulated pathways from TCGA and GEO datasets as well as the pathways from KEGG pathway and PPI network were further analyzed to filter two shared up-regulated pathways. Seven genes (COMP, COL10A1, COL11A1, COL3A1, THBS2, and COL1A1) that were shared by both pathways were defined as hub genes. Next, the correlation among the seven key genes and clinicopathological features were evaluated. The expression of the seven key genes was validated using immunohistochemistry staining and qPCR assays. Then, we used CiiiDER to identify the common transcription factor binding sites of the seven hub genes. Further *in vitro* experiments were applied to validate the role of CTCF in tumorigenesis and regulating these key genes (**Figure 1**).

### Gene Expression Programs in Tumors Recapitulate Clinically Distinct ECM Subtypes

**Figure 2:**
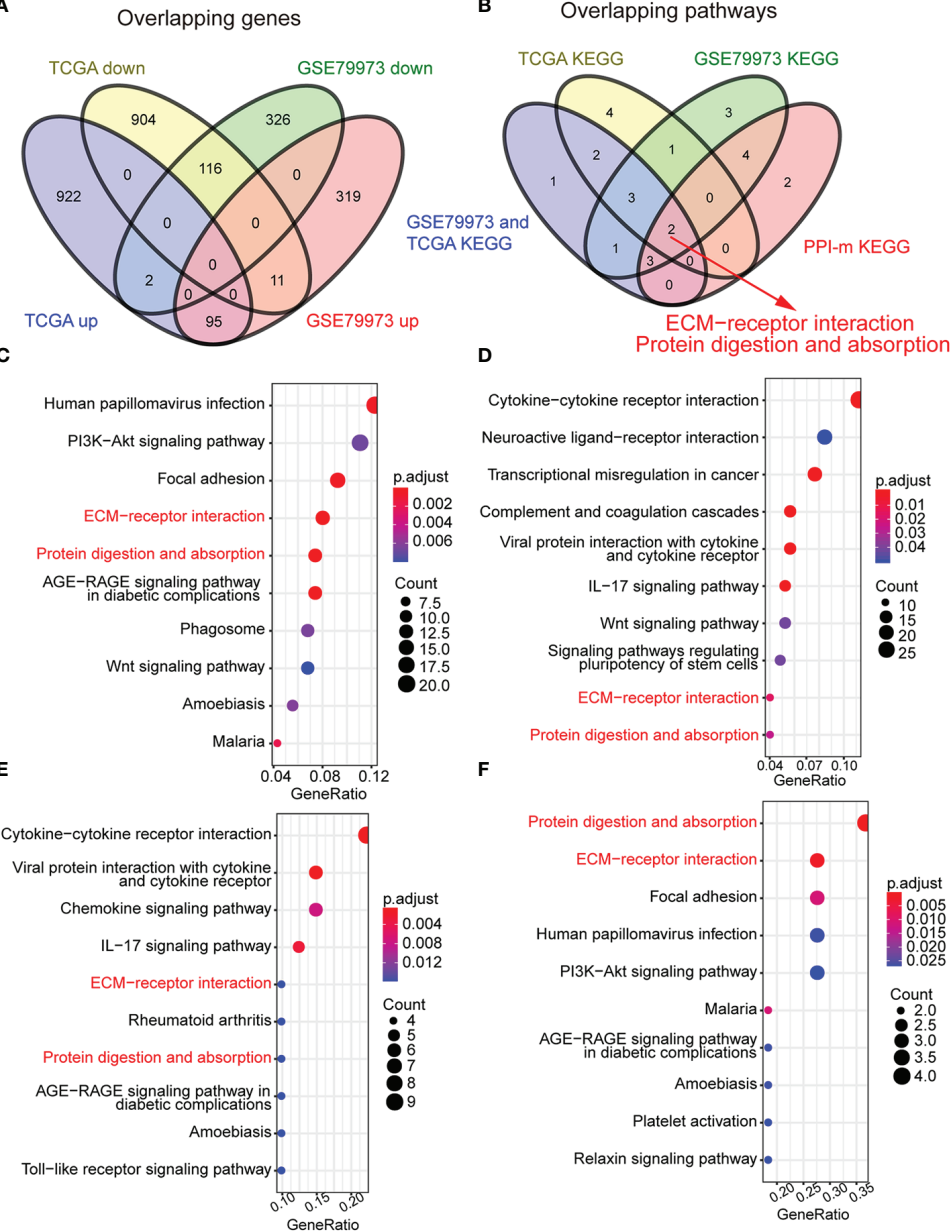
Identification of the extracellular matrix (ECM) subtype. **(A)** Venn diagrams of the differentially expressed genes (DEGs) between the GSE79973 dataset and the The Cancer Genome Atlas (TCGA) gastric cancer (GC)**** dataset. Ninety-five DEGs were upregulated and 116 DEGs were downregulated in the two datasets. **(B)** Venn diagrams of the overlapping pathways. A total of two enriched pathways overlapped the two datasets: ECM-receptor interaction and protein digestion and absorption. **(C)** Kyoto Encyclopedia of Genes and Genomes (KEGG) pathway enrichment analysis of the DEGs in the GSE79973 dataset. **(D)** KEGG pathway enrichment analysis of the DEGs in TCGA GC dataset. **(E)** KEGG pathway enrichment analysis of overlapping DEGs between the GSE79973 dataset and the TCGA dataset. **(F)** KEGG pathway enrichment analysis of the genes in the protein-protein interaction (PPI) module. The overlapping pathways are marked in red.

The GSE79973 gene expression profile and the TCGA GC dataset were used to identify the shared key genes and pathways that may play a pivotal role in the development of GC (**Supplementary Table 3**). A total of 869 DEGs were obtained from the GSE79973 dataset, which contains 10 gastric tumor samples and 10 paired adjacent normal samples. These 869 DEGs included 425 upregulated genes and 444 downregulated genes. The DEGs acquired from the TCGA GC dataset consisted of 1,019 upregulated and 1031 downregulated genes. A total of 221 DEGs were present in both the GSE79973 and TCGA GC datasets, including 95 upregulated genes and 116 downregulated genes (**Figure 2A**). To explore the potential biological effects of these genes, KEGG pathway analysis was conducted using the DEGs from each dataset. KEGG analysis showed that a total of 26 pathways were enriched in the GC group (**Figure 2B**). The DEGs from the GSE79973 dataset were significantly enriched in 10 pathways, including human papillomavirus infection, PI3K-Akt signaling, focal adhesion, ECM-receptor interaction, and protein digestion and absorption (**Figure 2C**, **Supplementary Table 4**). The top 10 upregulated enriched pathways from TCGA GC dataset included cytokine-cytokine receptor interaction, neuroactive ligand-receptor interaction, transcriptional dysregulation in cancer, complement, and coagulation cascades, viral protein interaction with cytokine and cytokine receptor, the IL-17 signaling pathway, the Wnt signaling pathway, signaling pathways regulating pluripotency of stem cells, ECM-receptor interaction and protein digestion and absorption (**Figure 2D**, **Supplementary Table 5**). We further assessed the pathways in which the 95 upregulated overlapping DEGs were enriched. Interestingly, the results indicated that the ECM-receptor interaction and protein digestion and absorption pathways were significantly upregulated in GC samples compared to normal samples (**Figure 2E**, **Supplementary Table 6**). These two pathways were also listed among the most enriched pathways in the individual GSE79973 and TCGA DEGs (**Figures 2C–D**).

To determine the potential interactions between these 95 upregulated overlapping genes in GC, we input these genes into the STRING website to construct a PPI network. The results showed that the upregulated DEGs formed a network with 92 nodes and 326 edges (**Supplementary Figure 1**). In total, two modules were enriched by protein–protein interaction network analysis, which included 20 genes (**Supplementary Figure 2**). KEGG pathway enrichment analysis of the genes from the PPI module revealed 10 upregulated enriched pathways (**Figure 2F**, **Supplementary Table 7**). Interestingly, ECM-receptor interaction and protein digestion and absorption were the only two shared up-regulated pathways across all the pathway analyses. By filtering the significant DEGs in these two pathways, a total of seven hub genes, including COL1A1, COL3A1, COL10A1, COL11A1, COMP, SPP1, and THBS2, were identified. These seven genes are all ECM-related genes.

### Elevated ECM Subtype Gene Expression is Significantly Correlated With Advanced Clinicopathological Indicators and Poor Prognosis

**Figure 3:**
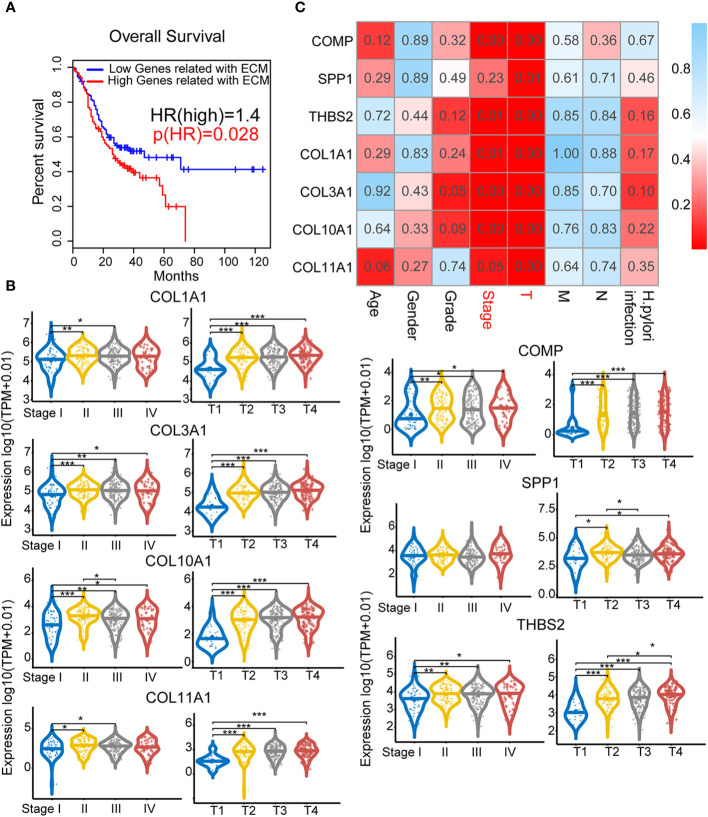
Clinical pathological indicators and prognostic characteristics of elevated extracellular matrix (ECM) subtype genes: Analysis of the correlations between ECM subtype gene expression and survival and clinical characteristics. **(A)** Survival analysis for the ECM subtype genes COMP, SPP1, THBS2, COL1A1, COL3A1, COL10A1, and COL11A1. **(B)** Correlations between the seven hub genes and clinical characteristics. The numbers in each small rectangle indicate the P-value for the correlation. **(C)** The relationship between the hub genes and clinical stage and T stage. *P < 0.05, **P < 0.01, ***P < 0.001.

We next evaluated whether these seven genes could be used as a gene signature for predicting prognostic results in GC patients. Based on the median gene expression, we divided all GC samples from the TCGA into two groups (high score versus low score). Intriguingly, GC patients with high gene signature score exhibited significantly (P=0.028) poorer survival outcomes compared to patients with low gene signature score (**Figure 3A**). We then asked whether each of these seven genes exhibited a high correlation with patients’ clinical features. As shown in **Figure 3B**, significantly higher expression of COL1A1, COL3A1, COL10A1, COL11A1, COMP, and THBS2 was observed in GC with advanced clinical and TNM stage compared with early-stage GC. In contrast, SPP1 expression only correlated with TNM stage, not clinical stage. This finding was further validated by one-way ANOVA in **Figure 3C**. The expression of these genes only significantly correlated with clinical stage and T stage but did not significantly correlate with patients’ age, gender, grade, M/N stage or *H. pylori* infection status.

### Validation of the Expression of ECM-Related Genes at the Protein Level Between GC Tissues and Normal Tissues and at the Transcriptional Level Between Aggressive and Less Aggressive GC Cells

**Figure 4:**
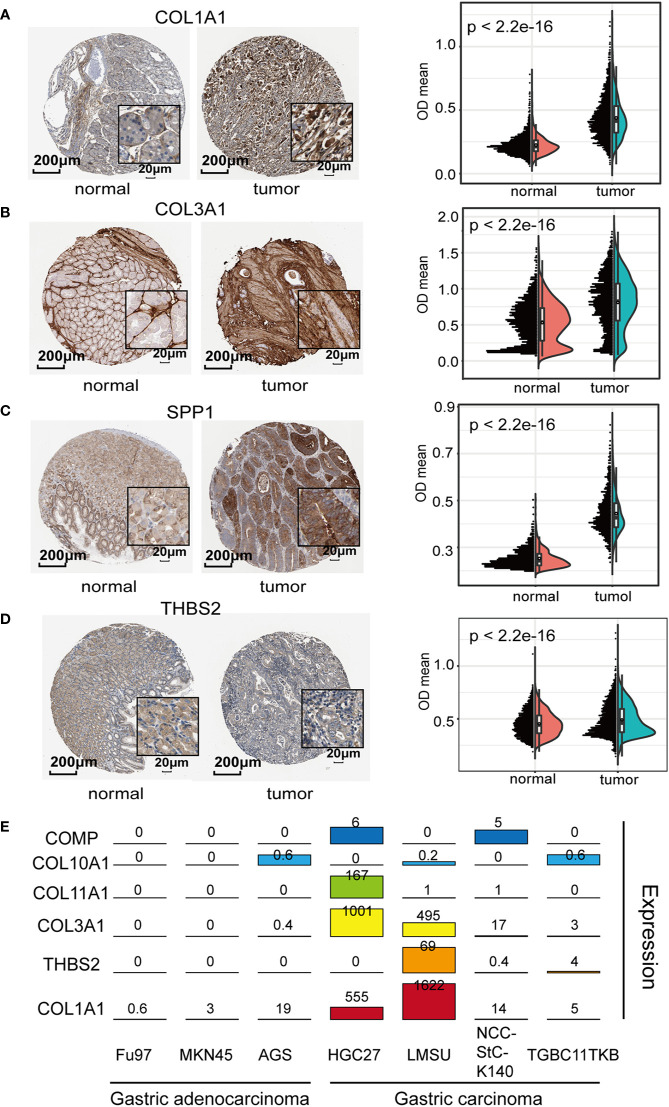
Exploration of the biological relevance of expression differences in the extracellular matrix (ECM) subtype genes: **(A–D)** Representative immunohistochemistry (IHC) images of ECM subtype genes in gastric cancer (GC) tissues and normal tissues [Human Protein Atlas (HPA) database]. The quantification results suggested that the protein levels of COL1A1, COL3A1, SPP1, and THBS2 were significantly increased in GC tumor tissues compared to normal gastric tissues. **(E)** Expression levels of the ECM subtype genes were determined in seven GC cell lines (aggressive cell lines: BGC823, HGC27, LMSU, and NCCStCK140; and less aggressive cell lines: FU97, MKN45, and AGS).

To validate the expression of the 7 genes in GC samples, we further confirmed the IHC staining results of COL1A1, COL3A1, SPP1, and THBS2 in both GC tumor and adjacent normal tissues using the HPA website (**Figures 4A–D**). The quantification results suggested that COL1A1, COL3A1, SPP1, and THBS2 protein levels were significantly increased in GC tumor tissues compared to normal adjacent tissues. We further investigated the mRNA levels of these genes in seven different GC cell lines (HGC27, TGBC11TKB, LMSU, NCC-StC-K140, FU97, MKN45, and AGS). The mRNA expression levels of these genes were significantly higher in the highly aggressive gastric carcinoma cell lines TGBC11TKB, LMSU, NCC-StC-K140, and HGC27 compared with the less aggressive adenocarcinoma cell lines FU97, MKN45 and AGS (**Figure 4E**). Overall, our results suggested that the transcript and protein expression levels of ECM subtype genes were upregulated in GC tumor samples, which validated our findings from the above analyses.

### ECM Subtype Genes Exhibit Unique CTCF Binding Motif and Histone Modification Patterns

**Figure 5:**
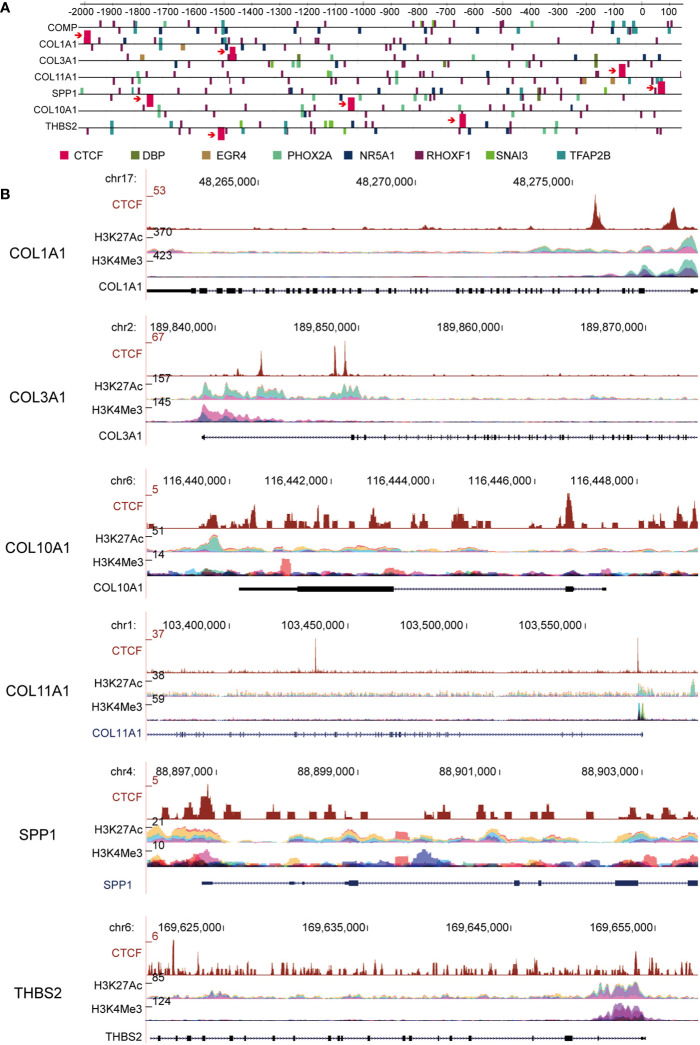
CCCTC-binding factor (CTCF) transcription factor binding motifs are present in a subset of extracellular matrix (ECM) candidate genes. **(A)** Computational predictions of binding sites of CTCF and other transcription factors are shown upstream of the ECM-related gene transcription factor binding sites (TFBSs); COL1A1, COL3A1, and COL11A1 genes have a common transcription factor: CTCF. **(B)** ChIP-Seq data of two common active markers, H3K27Ac and H3K4me3, in the promoter region of ECM subtype genes that overlapped with the CTCF binding sites.

CTCF is a well-known transcription factor that is essential in organizing chromatin and regulates target genes related to cancer (29). To explore the upstream transcription factors of the above hub genes, CiiiDER, a tool for predicting and analyzing transcription factor binding sites, was utilized to analyze mutual transcription factors of these hub genes. CiiiDER operates through an intuitive graphical user interface with interactive, high-quality visual output that makes it accessible to all researchers. CiiiDER predicts transcription factor binding sites (TFBSs) in different regulatory regions, such as promoters and enhancers from any species. CiiiDER can be used to identify TFs that are significantly under- or overexpressed compared to customized background sets; thus, it is useful to identify gene sets of pathophysiological importance (22). We scanned 2 kb upstream and 500 bp downstream from the transcription start site (TSS) for CTCF binding sites. Overlapping genes, including COL1A1, COL3A1, COL10A1, COL11A1, COMP, SPP1, and THBS2, were analyzed to identify common regulatory transcription factors based on three criteria: an upstream scan limit=2,000 bases, a downstream scan limit=500 bases, and deficit threshold=0.15. Moreover, the profiles of the transcription factor binding sites were retrieved from the JASPER database (**Figure 5A**). Given that these six key genes were significantly upregulated in GC, we wanted to understand whether the active histone modifications were associated with these genes. CHIP-Seq data of two common active markers, H3K27Ac and H3K4me3, were visualized using the ENCODE platform (**Figure 5B**). The results confirmed that H3K27Ac and H3K4me3 peaks were present in the promoter regions of COL1A1, COL3A1, COL10A1, COL11A1, SPP1, and THBS2 and overlapped with CTCF binding sites, indicating that the upregulation of these genes might be associated with H3K27Ac and H3K4me3.

### CTCF Expression Was Correlated With the Wnt Signaling Pathway

**Figure 6:**
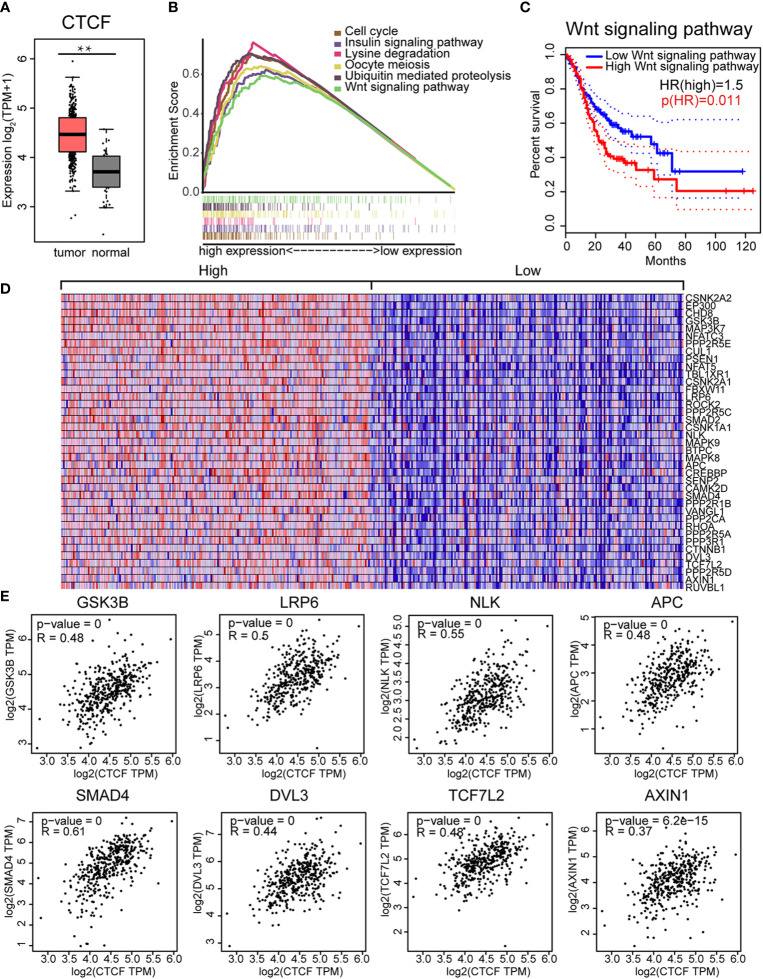
CCCTC-binding factor (CTCF) expression correlated with the Wnt signaling pathway: **(A)** The CTCF transcript was highly expressed in GC tissues compared with normal tissues. **(B)** The top six enriched pathways in GC samples with high CTCF expression were identified by gene set enrichment analysis (GSEA). **(C)** The prognostic value of the Wnt signaling pathway in gastric cancer (GC). Kaplan-Meier curves demonstrate the correlation between Wnt signaling protein expression and overall survival. **(D)** Gene Set Enrichment Analysis (GSEA)-generated heat map for highly enriched genes in samples with high CTCF expression vs. those with low CTCF expression. **(E)** Pearson’s correlation analysis of CTCF and Wnt pathway-related genes. We used the service provided by the website Gene Expression Profiling Interactive Analysis (GEPIA) (http://gepia.cancer-pku.cn/index.html) to examine the correlations among GSK3B, LRP6, NLK, APC, SMAD4, DVL3, TCF7L2, AXIN1, and CTCF.

CTCF expression in GC was assessed using Gene Expression Profiling Interactive Analysis (GEPIA) and verified with the TCGA database. CTCF was significantly overexpressed in GC tissues compared to adjacent normal tissues (**Figure 6A**). To explore the molecular mechanisms of CTCF in GC, ssGSEA analysis was performed to identify the enriched pathways related to CTCF. The top six enriched pathways were cell cycle, ubiquitin-mediated proteolysis, oocyte meiosis, insulin signaling, lysine degradation, and Wnt signaling (**Figure 6B**, **Supplementary Table 8**). In addition, we analyzed the DEGs in the whole Wnt signaling pathway and classified the GC patients into two groups. We found that patients with low expression (relative to the median) of Wnt signaling pathway proteins exhibited significantly better survival outcome compared with those with high expression (**Figure 6C**). Therefore, the Wnt signaling pathway could be an important pathway involved in the progression of GC. The heatmaps in **Figure 6D** illustrated the expression levels of individual genes involved in the Wnt pathway correlated to high CTCF expression. Further analyses showed CTCF expression was correlated with some keys genes in the Wnt pathways, including GSK3B, LRP6, NLK, APC, SMAD4, DVL3, TCF7L2, and AXIN1 (**Figure 6E**). The top three genes co-expressed with CTCF were (a) SMAD4 (R = 0.61), (b) NLK (R = 0.55), and (c) LRP6 (R = 0.5) (**Figure 6E**). Our data shown the CTCF expression correlated APC expression in the one of most affected pathways.

### CTCF Regulates ECM-Related Genes and Contributes to Promoting Cell Growth and Migration in GC Cell Lines

**Figure 7:**
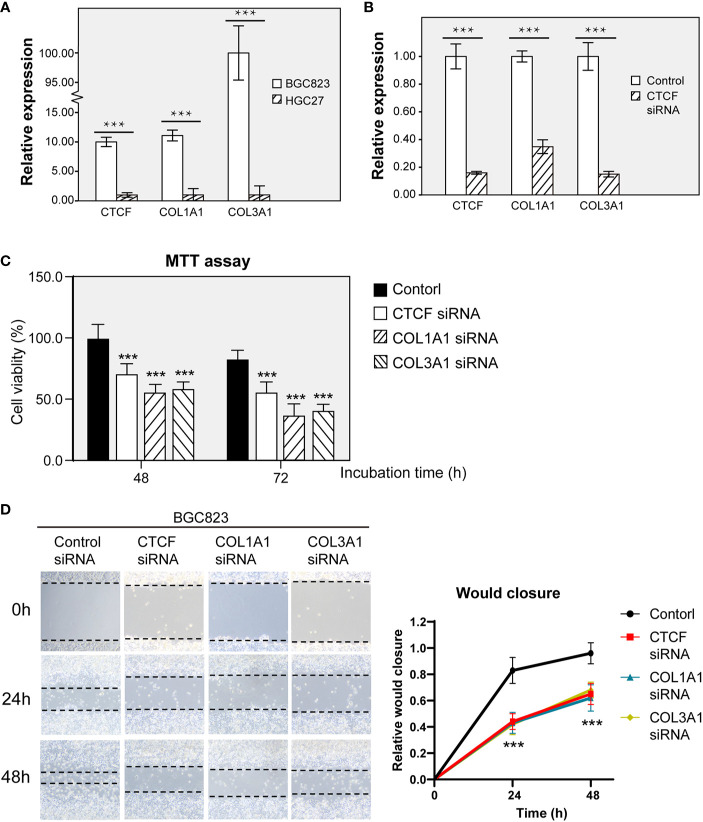
CTCF, COL1A1, and COL3A1 promote gastric cancer (GC) cell viability and migration *in vitro*. **(A)** Comparison of CTCF, COL1A1, and COL3A1 mRNA expression between BGC823 and HCG27 by RT-qPCR. **(B)** Real-time qRT-PCR was performed to detect the expression of the indicted genes to verify the effectiveness of knockdown. **(C)** BGC823 cells were treated with siRNA for 24, 48, and 72 h, and the MTT index was measured based on the MTT assay. The result is presented as fold change of metabolic viability/cell and normalized with respect to control. **(D)** Wound−healing assays were used to determine the effects of single siRNAs targeting CTCF, COL1A1, or COL3A1 on BGC823 cell migration. The bar chart represents the wound width (%) at 24 or 48 h divided by the width at 0 h. Data are presented as the mean ± SD of three independent experiments. **P < 0.001.

The ECM regulates diverse cellular functions, including cell survival, migration, and dysregulation of the ECM could lead to cancer progression (30). First, we selected the human gastric cell lines BGC-823 and HGC27 based on their cell migration, invasion and epithelial-mesenchymal transition (EMT) features to represent aggressive tumor cell lines. To evaluate the potential clinical relevance of CTCF and these ECM-related genes, we first examined the mRNA expression of CTCF and other two key ECM-related genes COL1A1 and COL3A1 in BGC823 and HGC27 human GC cell lines (**Figure 7A**). Given that the BCG823 cell line exhibited increased CTCF, COL1A1, and COL3A1 expression, we generated single siRNA to interfere with CTCF signaling in the BGC823 cell line. CTCF siRNA interference not only significantly reduced CTCF mRNA levels but also resulted in reduction of COL1A1 and COL1A3 mRNA expression in BCG823 cells (**Figure 7B**). This finding further demonstrated that CTCF might regulate COL1A1 and COL1A3 expression in GC cells *in vitro*. To explore the cellular function of CTCF, COL1A1, and COL1A3, we further used siRNA to interfere with CTCF, COL1A1, and COL1A3 expression, respectively, in BGC823 cells. MTT assay results demonstrated that BGC823 cell viability was significantly decreased in cells transfected with CTCF siRNA, COL1A1 siRNA or COL1A3 siRNA when compared with siRNA control cells after 48 or 72 h (**Figure 7C**). Similarly, the migration ability of cells transfected with CTCF siRNA, COL1A1 siRNA, or COL1A3 siRNA was decreased compared to siRNA control cells (**Figure 7D**). Overall these results indicated that CTCF could regulate the expression of COL1A1 and COL1A3 *in vitro* and silencing CTCF, COL1A1, and COL3A1 expression led to decreased cell viability and migration in BGC823 cells.

## Discussion

GC is a leading cause of cancer-related death due to a lack of knowledge about the relevant molecular classification of subtypes in pathogenic development. Thus, identifying key transcription factors that regulate the molecular signature associated with GC progression will definitely be useful. Here, an integrative strategy was used to comprehensively analyze the variable patterns of GC based on RNA-Seq, ChIP-Seq, gene profiling and molecular signature studies. We discovered a key molecular subtype, namely, a 7-gene signature related to ECM subtype that is linked to distinct patterns of molecular alterations and to disease progression and prognosis. In addition, ECM-related genes in this subtype are regulated by an important transcription factor, the zinc finger protein CTCF. Furthermore, we validated the expression of proteins and mRNAs related to CTCF and the ECM in tumor tissues and in aggressive and less aggressive GC cell lines. We found that the silencing of CTCF decreased mRNA expression of COL1A1 and COL1A3, two key ECM-related genes included in the 7-gene signature. Furthermore, silencing CTCF and two ECM-related genes caused reductions in cell viability and migration. These results reveal that the ECM-related 7-gene signature could be a useful prognostic factor for survival. The zinc finger protein CTCF regulates ECM-related genes and plays an important role in promoting GC cell growth and migration *in vitro*.

The combination of ChIP-Seq and transcriptomic analysis is gradually becoming the standard for molecular spectrum analysis of various cancers. The challenge is now to combine these different molecular biological data to obtain a more comprehensive and informative view of key biological processes in cancer progression. Our objective was to systematically analyze RNA-Seq, ChIP-Seq, gene map, and molecular marker data in GC to understand the major regulatory factors and target genes in this disease. Our study identified differences in mRNA expression between tumors and adjacent normal tissues from different clinical cohorts. The identified DEGs were filtered through KEGG, PPI network and functional pathway analyses. We were the first to identify a key molecular subtype, referred to as the ECM subtype, and provide prognostic factors. The expression of ECM-related genes was also correlated with the clinical status of cancer progression. GC pathway analysis results were consistent with known processes in GC, such as ECM-receptor interaction and protein digestion and absorption. Furthermore, our ChIP-Seq and histone modification analysis results suggested that the expression of these target ECM-related genes is influenced by the distance from the transcription start site (TSS) to their CTCF binding sites. Of note, KEGG pathway analysis identified transcription factor binding site motifs and histone modification markers, demonstrating that DEGs with CTCF-binding motif modifications exhibiting functional enrichment during cancer development. The expression of these target genes is closely related to OS in GC patients. Kaplan-Meier survival analysis confirmed this findings (**Figures 3** and **6**). We found that CTCF may modulate previously unreported ECM target genes and mediate their possible roles. In summary, combining data from different sources is a promising strategy for identifying major regulators and target genes for cancer.

The chromatin‐organizing CCCTC‐binding factor (CTCF) encodes a transcription regulator protein with 11 highly conserved zinc finger domains that binds to more than 20,000 DNA loci in the human genome (31). CTCF plays indispensable roles in transcriptional inhibition/activation, insulation, gene imprinting, and regulation of 3D chromatin structure (32). Studies have previously demonstrated that chromosomal architecture is largely mediated by the CTCF/cohesin complex (33, 34) because it is of vital importance in promoting the formation of cohesin-mediated loops and boundaries that are necessary for gene regulation. This process enables the organization of chromatin into highly self-interacting topologically associated domains (10). Its expression pattern and roles in different tumor types vary greatly. For instance, CTCF overexpression was previously reported in breast cancer (11), cervical cancer (12), ovarian cancer (13), and hepatocellular carcinoma (14) and is often related to tumor features with adverse prognostic impacts. Additionally, mutations in CTCF or defects in CTCF function have been observed in GC (16). However, the clinical impact of CTCF expression in GC is unclear. In this study, we identified CTCF as an important contributor to GC progression, survival and prognosis. Then, we conducted ssGSEA to perform hallmark analysis, which defines an enrichment score that represents the degree of absolute enrichment of a gene set in each sample within a given dataset (35). This analysis demonstrated that the Wnt/signaling pathway was a key pathway that was highly related to CTCF.

Here, we sought to determine whether CTCF was regulated by a signaling pathway or associated with another pathway involved in gastric cancer. The Wnt signal transduction pathway is a highly conserved extracellular signal transduction pathway that is implicated in many vital cellular functions, such as stem cell regeneration and organogenesis. Several intra-cellular signal transduction pathways are induced by Wnt, notably the Wnt/beta-catenin dependent pathway, which is known as the canonical pathway, and the non-canonical or beta-catenin-independent pathway. The latter includes the Wnt/Ca^2+^ and Planar Cell Polarity pathway (PCP) (36–39). Colorectal cancers exhibit evidence of Wnt signaling pathway activation associated with loss of function of the tumor regulator APC. Our data also demonstrated that CTCF expression correlated with APC expression in the one of most affected pathways (**Figure 6**). This finding indicated the some ECM genes are linked to the Wnt signaling pathway through the CTCF transcription factor. Taken together, these data confirm that Wnt signaling is an important mechanism driving GC metastasis and can therefore be considered an attractive therapeutic target.

Major efforts have been made to elucidate the molecular mechanisms that regulate the Wnt signaling pathway and its role in development, homeostasis, and cancer. However, there is a lack of understanding about the relationships between Wnt signaling and ECM adhesion. WNT7A depletion in 5637 HMI and T24 cells reduced urinary bladder cancer (UBC) cell invasion and decreased the levels of active β-catenin and its downstream target genes involved in ECM degradation, but these studies lacked detail on the regulatory mechanism between Wnt and ECM. ECM is an important component of the tumor microenvironment and plays critical roles in cancer development and metastasis, in which collagen is the major structural protein. Collagen type I alpha 1 (COL1A1) reportedly promotes cancer cell migration. In our study, knockdown of COL1A1 or COL3A1 inhibited GC cell metastasis with low levels of CTCF and WNT5 or CTCF and WNT3 (**Supplementary Figure 3**), which is independent of the epithelial-mesenchymal transition (EMT) process. Our results have identified a CTCF/WNT axis that controls GC migration through ECM regulation *via* the canonical Wnt/β-catenin signaling, which may offer prognostic and therapeutic opportunities.

There are several limitations in this study. First, although the Wnt signaling pathway was highly correlated with CTCF expression, we did not have sufficient experimental evidence to elucidate the detailed relationships between CTCF and the Wnt signaling pathway. Second, more experiments were needed to double-confirm whether CTCF could regulate the expression of other ECM-related genes besides COL1A1 and COL1A3. Protein level validations should be performed. Third, more experiments were needed to determine whether CTCF-mediated regulation of COL1A1/COL1A3 occurred directly or indirectly.

## Conclusion

In the present study, we systematically analyze the RNA-Seq, ChIP-Seq, gene profiling, and molecular signature data of GC and performed experimental validation to obtain insight into the main regulators and target genes in GC. We identified that the zinc finger protein CTCF regulates the ECM-related genes COL1A1 and COL1A3 *via* a novel mechanism and that this pathway could be used to evaluate GC progression. Overall, using a combination of multiple omics analyses and wet lab experimental data, the present study provides a potential prognostic factor for survival prediction among GC patients.

## Data Availability Statement

The datasets presented in this study can be found in online repositories. The names of the repository/repositories and accession number(s) can be found in the article/**Supplementary Material**.

## Ethics Statement

Ethical review and approval were not required for the study on human participants in accordance with the local legislation and institutional requirements. Written informed consent for participation was not required for this study in accordance with the national legislation and the institutional requirements. Written informed consent was not obtained from the individual(s) for the publication of any potentially identifiable images or data included in this article.

## Author Contributions

YH, LY, and DX made a substantial contribution to the concept and design of the work. CL, LD, JiaZ, JL, PJ, JinZ, and SH performed the acquisition, analysis, and interpretation of the data. CL, LD, YH, LY, and DX drafted the article. PX, PN, LY, DX, and YH revised it critically for important intellectual content. All authors contributed to the article and approved the submitted version.

## Funding

This work was supported by grants from the National Natural Science Foundation of China (81871274, 31670905, 81871715 and 82071811).

## Conflict of Interest

The authors declare that the research was conducted in the absence of any commercial or financial relationships that could be construed as a potential conflict of interest.
