# Supplementary material for: Zinc Finger Protein CTCF Regulates Extracellular Matrix (ECM)-Related Gene Expression Associated With the Wnt Signaling Pathway in Gastric Cancer
Source: Front Oncol. 2021 Feb 16;10:625633. doi: 10.3389/fonc.2020.625633 (PMC7921701; doi:10.3389/fonc.2020.625633)
Supplement: Supplementary file 1 [file DataSheet_1.docx]

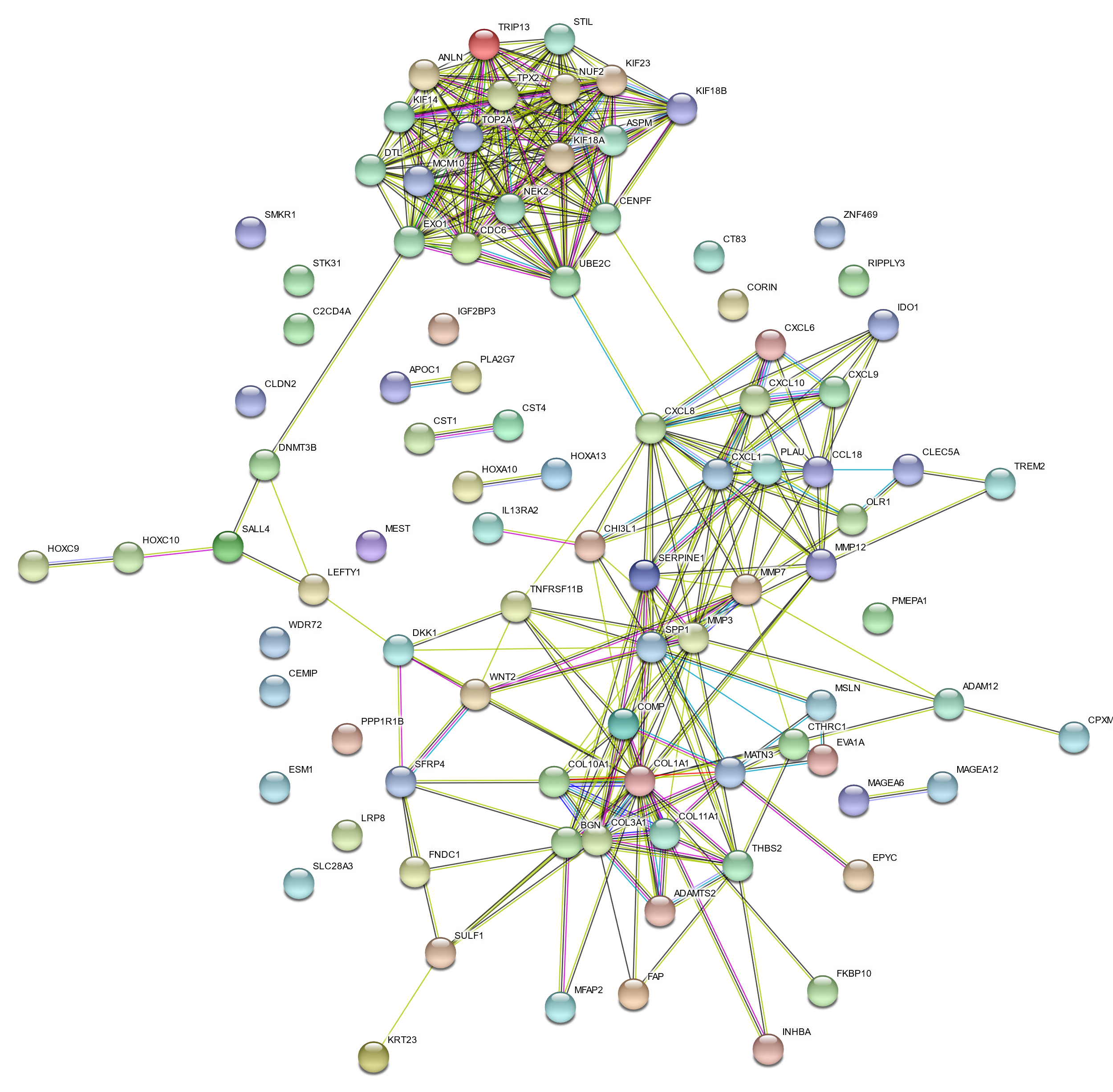


**Supplementary Figure 1.** Protein-PPI network analysis of 95 up-regulated overlapping genes in GC. Each circle represents a node and each line represents an edge. The different color of the lines represents different types of interactions: light blue: curated databases; pink: experimentally determined; green: gene neighborhood; red: gene fusions; blue: gene co-occurrence; yellow: textmining; dark: co-expression; grey: protein homology.


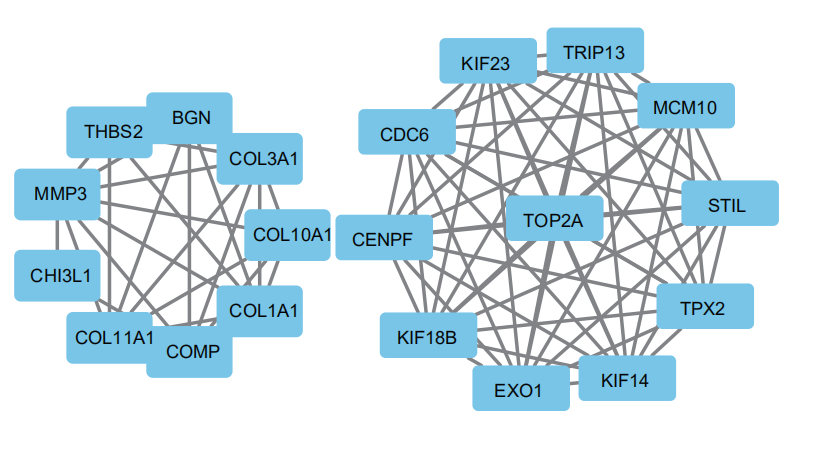


**Supplementary Figure 2.** Protein–protein interaction network analysis of Overlapping genes. Each square represents a node and each line represents an edge.


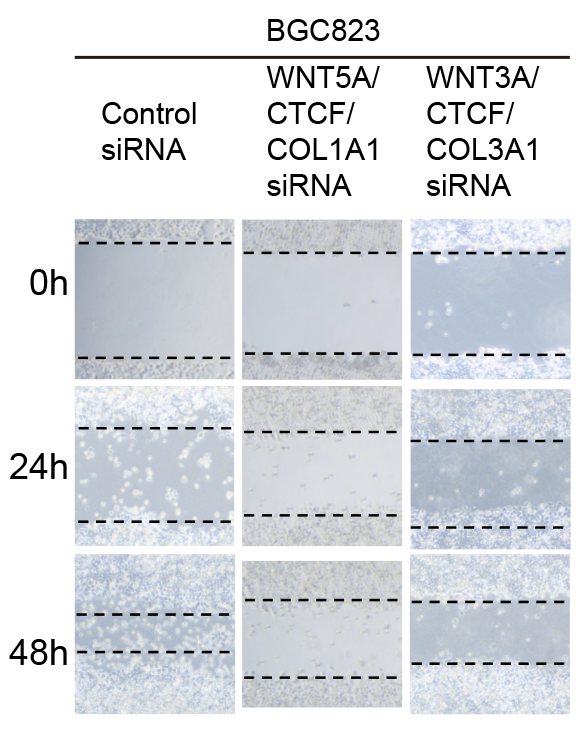


­­**Supplementary Figure 3.** Migratory ability assay of combine multiples of siRNA for WNT5A/CTCF/COL1A1, WNT3A/CTCF/COL3A1 for BGC823 cells.
