# Supplementary material for: Zinc Finger Protein CTCF Regulates Extracellular Matrix (ECM)-Related Gene Expression Associated With the Wnt Signaling Pathway in Gastric Cancer
Source: Front Oncol. 2021 Feb 16;10:625633. doi: 10.3389/fonc.2020.625633 (PMC7921701; doi:10.3389/fonc.2020.625633)
Supplement: Supplementary file 2 [file DataSheet_2.docx]

**Table S1 The siRNA sequences targeting CTCF, COL1A1, COL3A1 and the Negative control siRNA sequences**

|  | Sequences |
| --- | --- |
| CTCF | 5’-GUGCAAUUGAGAACAUUAUTT-3’,  5’-GAUGAAGACUGAAGUAAUGTT-3’,  5’- GGAGAAAGAAGAAGAGUATT-3’,  5’-GAAGAUGCCUGCCACUUACTT-3’,  5’-GAACAGCCCAUAAACAUAGTT-3’ |
| COL1A1 | 5’-GAAGGGCCAAGCACGACAATT-3’,  5’-GAAGGGCCACGACAAAGCATT-3’ |
| COL3A1 | 5’-GAUGCUAUCAAGGUAUUCUTT-3’,  5’-GGAUGCAAAUUGGAUGCUATT-3′,  5’-CCCUCCUAAUGGUCAAGGATT-3′ |
| Control | 5′-UUCUCCGAACGUGUCACGU-3′ |

**Table S2 Primers information of ECM subtype genes**

| Gene name | Primers sequences |
| --- | --- |
| COMP | F: 5’-AACTCAGGGCAGGAGGATGT |
|  | R: 5’-TGTCCTTTTGGTCGTCGTTC |
| COL10A1 | F: 5’-GGACTCTCAACTTCCCACCTTT |
|  | R: 5’-AAACACTTTCCCTCAAAGGTGGA |
| COL11A1 | F: 5’-TTGATGTTACCGTTCCGTTATG |
|  | R: 5’-AATCCGAGCCTGCTGAAGAAT |
| COL1A1 | F: 5’-CTGCTGGACGTCCTGGTGAA |
|  | R: 5’-ACGCTGTCCAGCAATACCTTGAG |
| COL3A1 | F: 5’-TTGAAGGAGGATGTTCCCATCT |
|  | R: 5’-ACAGACACATATTTGGCATGGTT |
| THBS2 | F: 5’-CGTGGACAATGACCTTGTTG |
|  | R: 5’-GCCATCGTTGTCATCATCAG |

**Table S3 Information of the data resources**

| Groups | Gastric mucosa  (number of samples) | Gastric adenocarcinoma  (number of samples) | Experiment type |
| --- | --- | --- | --- |
| TCGA  (TCGA-STAD) ^#^ | 32 | 275 | RNA-sequencing |
| GEO DataSet  (GSE79973) ^1, 2^* | 10 | 10 | Micro array(mRNA) |

Abbreviations: TCGA: The Cancer Genome Atlas

#TCGA-STAD: project ID of the data we used from TCGA

*The dataset we used for validation also can be searched in GEO(Gene Expression Omnibus microarray data repository), GEO accessions and references were shown.

**References:**

1. He, J.; Jin, Y.; Chen, Y.; Yao, H. B.; Xia, Y. J.; Ma, Y. Y.; Wang, W.; Shao, Q. S., Downregulation of ALDOB is associated with poor prognosis of patients with gastric cancer. *Onco Targets Ther* **2016,** *9*, 6099-6109.

2. Jin, Y.; He, J.; Du, J.; Zhang, R. X.; Yao, H. B.; Shao, Q. S., Overexpression of HS6ST2 is associated with poor prognosis in patients with gastric cancer. *Oncol Lett* **2017,** *14* (5), 6191-6197.

**Table S4 Up-regulated KEGG pathway of differentially expressed genes in GSE79973**

| ID | Description | GeneRatio | pvalue | qvalue | geneID |
| --- | --- | --- | --- | --- | --- |
| hsa04512 | ECM-receptor interaction | 13/165 | 2.27E-08 | 3.69E-06 | THBS2,SPP1,THBS4,COL1A2,COL6A3,FN1,COL4A1,COMP,COL1A1,COL4A2,TNC,THBS1,ITGA2 |
| hsa04974 | Protein digestion and absorption | 12/165 | 4.71E-07 | 3.82E-05 | COL10A1,COL11A1,COL1A2,COL5A2,COL6A3,COL12A1,COL4A1,COL5A1,COL3A1,COL1A1,COL18A1,COL4A2 |
| hsa04933 | AGE-RAGE signaling pathway in diabetic complications | 12/165 | 8.28E-07 | 4.48E-05 | COL1A2,CXCL8,FN1,COL4A1,NOX4,SELE,VEGFC,COL3A1,COL1A1,COL4A2,IL6,SERPINE1 |
| hsa05165 | Human papillomavirus infection | 20/165 | 1.27E-05 | 0.000429 | THBS2,SPP1,THBS4,COL1A2,COL6A3,WNT2,FN1,COL4A1,COMP,PTGS2,COL1A1,COL4A2,PDGFRB,WNT5A,TNC,THBS1,FZD7,HEY1,CCNA2,ITGA2 |
| hsa04510 | Focal adhesion | 15/165 | 1.32E-05 | 0.000429 | THBS2,SPP1,THBS4,COL1A2,COL6A3,FN1,COL4A1,COMP,VEGFC,COL1A1,COL4A2,PDGFRB,TNC,THBS1,ITGA2 |
| hsa05144 | Malaria | 7/165 | 6.44E-05 | 0.00174 | THBS2,THBS4,CXCL8,SELE,COMP,IL6,THBS1 |
| hsa05146 | Amoebiasis | 9/165 | 0.000233 | 0.005403 | COL1A2,CXCL8,FN1,COL4A1,COL3A1,COL1A1,CXCL1,COL4A2,IL6 |
| hsa04145 | Phagosome | 11/165 | 0.000279 | 0.005659 | THBS2,THBS4,OLR1,COMP,TUBB6,CTSL,C3,TUBA1A,NCF2,THBS1,ITGA2 |
| hsa04151 | PI3K-Akt signaling pathway | 18/165 | 0.00033 | 0.005947 | THBS2,SPP1,THBS4,COL1A2,COL6A3,FN1,COL4A1,COMP,VEGFC,COL1A1,COL4A2,IL6,PDGFRB,TNC,THBS1,ANGPT2,ITGA2,OSMR |
| hsa04310 | Wnt signaling pathway | 11/165 | 0.000434 | 0.007043 | SFRP4,MMP7,WNT2,CCN4,DKK1,SFRP2,RUVBL1,WNT5A,FZD7,SERPINF1,DKK2 |
| hsa04657 | IL-17 signaling pathway | 8/165 | 0.000664 | 0.009778 | CXCL8,CXCL6,PTGS2,CXCL1,IL6,MMP1,CXCL10,CEBPB |
| hsa04110 | Cell cycle | 9/165 | 0.000984 | 0.012671 | CDC6,CDC25B,CDC20,CCNB1,MAD2L1,BUB1B,CCNA2,TTK,CDC7 |
| hsa05205 | Proteoglycans in cancer | 12/165 | 0.001016 | 0.012671 | COL1A2,WNT2,FN1,LUM,PLAU,COL1A1,CTSL,TWIST1,WNT5A,THBS1,FZD7,ITGA2 |
| hsa04668 | TNF signaling pathway | 8/165 | 0.002076 | 0.02404 | SELE,CXCL6,PTGS2,VEGFC,CXCL1,IL6,CXCL10,CEBPB |
| hsa05222 | Small cell lung cancer | 7/165 | 0.002749 | 0.029614 | FN1,COL4A1,PTGS2,COL4A2,CKS1B,CKS2,ITGA2 |
| hsa05323 | Rheumatoid arthritis | 7/165 | 0.002923 | 0.029614 | CXCL8,CXCL6,CXCL1,CTSL,IL6,MMP1,CTSK |
| hsa04061 | Viral protein interaction with cytokine and cytokine receptor | 7/165 | 0.004384 | 0.041801 | CXCL8,CCL18,CXCL6,CXCL1,IL6,CXCL9,CXCL10 |

**Table S5 Up-regulated KEGG pathway of differentially expressed genes in TCGA datasets**

| ID | Description | GeneRatio | pvalue | qvalue | geneID |
| --- | --- | --- | --- | --- | --- |
| hsa04060 | Cytokine-cytokine receptor interaction | 28/253 | 1.43e-07 | 3.09E-05 | CSF2,CCL7,IFNL3,IL37,INHA,IFNL2,INHBA,CXCL8,CXCL9,IL11,CXCL11,AMH,PPBP,TNFRSF11B,CXCL10,CXCL6,CXCL1,IL17C,GDF15,IL13RA2,TNFSF11,CXCL5,CCR8,LIF,CCL26,THPO,CCL18,CCL20 |
| hsa04610 | Complement and coagulation cascades | 14/253 | 3.49E-07 | 3.77E-05 | C4BPA,F2,CFHR4,FGB,KNG1,VTN,CFHR5,CPB2,PLG,SERPIND1,CFHR3,SERPINE1,PLAU,F5 |
| hsa04061 | Viral protein interaction with cytokine and cytokine receptor | 14/253 | 2.66E-06 | 0.000191229 | CCL7,IL37,CXCL8,CXCL9,CXCL11,PPBP,CXCL10,CXCL6,CXCL1,CXCL5,CCR8,CCL26,CCL18,CCL20 |
| hsa05202 | Transcriptional misregulation in cancer | 19/253 | 5.81E-06 | 0.000301667 | SSX1,MMP3,HOXA11,CSF2,HMGA2,MPO,HOXA10,CXCL8,HOXA9,PAX3,WT1,ETV4,PAX7,SIX1,ELANE,TLX1,FCGR1A,MET,PLAU |
| hsa04657 | IL-17 signaling pathway | 13/253 | 6.99E-06 | 0.000301667 | MMP3,CSF2,MMP13,CCL7,CXCL8,S100A7A,CXCL10,MAPK15,CXCL6,CXCL1,IL17C,CXCL5,CCL20 |
| hsa04512 | ECM-receptor interaction | 10/253 | 0.00042475 | 0.015276095 | IBSP,COMP,SPP1,VTN,COL1A1,DSPP,DMP1,THBS2,LAMC2,COL9A3 |
| hsa04974 | Protein digestion and absorption | 10/253 | 0.000783912 | 0.024165716 | COL10A1,COL11A1,CTRB2,CTRB1,COL1A1,COL22A1,CPB2,COL9A3,PRSS1,COL3A1 |
| hsa04550 | Signaling pathways regulating pluripotency of stem cells | 12/253 | 0.001524206 | 0.038387495 | LEFTY1,ESX1,WNT2,DUSP9,INHBA,HAND1,ONECUT1,LIF,WNT11,OTX1,POU5F1B,WNT8B |
| hsa04310 | Wnt signaling pathway | 13/253 | 0.001601039 | 0.038387495 | NOTUM,WNT2,CER1,PRKCG,MMP7,SFRP4,BAMBI,LGR5,RSPO4,NKD2,WNT11,DKK1,WNT8B |
| hsa04080 | Neuroactive ligand-receptor interaction | 21/253 | 0.002434928 | 0.046178897 | HTR2C,CALCA,F2,KNG1,GABRD,CHRNA1,PTH2R,NTS,APLN,GABRA3,GRM8,GRIN2D,GABRR1,PRSS1,NPFFR1,PLG,AGT,NTSR1,KISS1R,UCN2,UTS2 |
| hsa04062 | Chemokine signaling pathway | 14/253 | 0.002567648 | 0.046178897 | GNGT1,CCL7,CXCL8,CXCL9,CXCL11,PPBP,CXCL10,CXCL6,CXCL1,CXCL5,CCR8,CCL26,CCL18,CCL20 |
| hsa05323 | Rheumatoid arthritis | 9/253 | 0.002567997 | 0.046178897 | MMP3,CSF2,CXCL8,IL11,CXCL6,CXCL1,TNFSF11,CXCL5,CCL20 |

**Table S6 Up-regulated KEGG pathway of Overlapping genes**

| ID | Description | GeneRatio | pvalue | qvalue | geneID |
| --- | --- | --- | --- | --- | --- |
| hsa04061 | Viral protein interaction with cytokine and cytokine receptor | 6/41 | 1.02E-05 | 0.000435 | CXCL8,CXCL9,CXCL10,CXCL6,CXCL1,CCL18 |
| hsa04060 | Cytokine-cytokine receptor interaction | 9/41 | 1.31E-05 | 0.000435 | INHBA,CXCL8,CXCL9,TNFRSF11B,CXCL10,CXCL6,CXCL1,IL13RA2,CCL18 |
| hsa04657 | IL-17 signaling pathway | 5/41 | 0.000106 | 0.002352 | MMP3,CXCL8,CXCL10,CXCL6,CXCL1 |
| hsa04062 | Chemokine signaling pathway | 6/41 | 0.000357 | 0.005915 | CXCL8,CXCL9,CXCL10,CXCL6,CXCL1,CCL18 |
| hsa04512 | ECM-receptor interaction | 4/41 | 0.001003 | 0.012369 | COMP,SPP1,COL1A1,THBS2 |
| hsa05323 | Rheumatoid arthritis | 4/41 | 0.001233 | 0.012369 | MMP3,CXCL8,CXCL6,CXCL1 |
| hsa04974 | Protein digestion and absorption | 4/41 | 0.001335 | 0.012369 | COL10A1,COL11A1,COL1A1,COL3A1 |
| hsa04933 | AGE-RAGE signaling pathway in diabetic complications | 4/41 | 0.001614 | 0.012369 | CXCL8,COL1A1,COL3A1,SERPINE1 |
| hsa05146 | Amoebiasis | 4/41 | 0.001736 | 0.012369 | CXCL8,COL1A1,CXCL1,COL3A1 |
| hsa04620 | Toll-like receptor signaling pathway | 4/41 | 0.001865 | 0.012369 | SPP1,CXCL8,CXCL9,CXCL10 |
| hsa05144 | Malaria | 3/41 | 0.002054 | 0.012382 | COMP,CXCL8,THBS2 |
| hsa04668 | TNF signaling pathway | 4/41 | 0.002446 | 0.013519 | MMP3,CXCL10,CXCL6,CXCL1 |

**Table S7 Up-regulated KEGG pathway of genes in PPI module**

| ID | Description | GeneRatio | pvalue | qvalue | geneID |
| --- | --- | --- | --- | --- | --- |
| hsa04974 | Protein digestion and absorption | 4/11 | 5.71E-06 | 5.41E-05 | COL1A1,COL3A1,COL10A1,COL11A1 |
| hsa04512 | ECM-receptor interaction | 3,11 | 0.000197 | 0.000935 | COL1A1,COMP,THBS2 |
| hsa05144 | Malaria | 2,11 | 0.002019 | 0.005067 | COMP,THBS2 |
| hsa04510 | Focal adhesion | 3,11 | 0.002139 | 0.005067 | COL1A1,COMP,THBS2 |
| hsa04933 | AGE-RAGE signaling pathway in diabetic complications | 2,11 | 0.007858 | 0.012029 | COL1A1,COL3A1 |
| hsa05146 | Amoebiasis | 2,11 | 0.008165 | 0.012029 | COL1A1,COL3A1 |
| hsa05165 | Human papillomavirus infection | 3,11 | 0.008888 | 0.012029 | COL1A1,COMP,THBS2 |
| hsa04151 | PI3K-Akt signaling pathway | 3,11 | 0.01078 | 0.01215 | COL1A1,COMP,THBS2 |
| hsa04611 | Platelet activation | 2,11 | 0.011891 | 0.01215 | COL1A1,COL3A1 |
| hsa04926 | Relaxin signaling pathway | 2,11 | 0.012825 | 0.01215 | COL1A1,COL3A1 |
| hsa04145 | Phagosome | 2,11 | 0.017523 | 0.015092 | COMP,THBS2 |

**Table S8 Up-regulated GSEA pathways**

| NAME | SIZE | ES | NES | NOM p-val | FDR q-val |
| --- | --- | --- | --- | --- | --- |
| KEGG_CELL_CYCLE | 124 | 0.7 | 2.07 | 0 | 0.038 |
| [KEGG_UBIQUITIN_MEDIATED_PROTEOLYSIS](http://www.gsea-msigdb.org/gsea/msigdb/cards/KEGG_UBIQUITIN_MEDIATED_PROTEOLYSIS) | 134 | 0.71 | 2.06 | 0 | 0.02 |
| [KEGG_OOCYTE_MEIOSIS](http://www.gsea-msigdb.org/gsea/msigdb/cards/KEGG_OOCYTE_MEIOSIS) | 112 | 0.64 | 2.06 | 0 | 0.014 |
| [KEGG_INSULIN_SIGNALING_PATHWAY](http://www.gsea-msigdb.org/gsea/msigdb/cards/KEGG_INSULIN_SIGNALING_PATHWAY) | 137 | 0.62 | 2.06 | 0 | 0.012 |
| [KEGG_LYSINE_DEGRADATION](http://www.gsea-msigdb.org/gsea/msigdb/cards/KEGG_LYSINE_DEGRADATION) | 44 | 0.76 | 2.04 | 0 | 0.01 |
| [KEGG_WNT_SIGNALING_PATHWAY](http://www.gsea-msigdb.org/gsea/msigdb/cards/KEGG_WNT_SIGNALING_PATHWAY) | 150 | 0.59 | 2.04 | 0 | 0.009 |
| [KEGG_GLYCEROPHOSPHOLIPID_METABOLISM](http://www.gsea-msigdb.org/gsea/msigdb/cards/KEGG_GLYCEROPHOSPHOLIPID_METABOLISM) | 77 | 0.6 | 2.03 | 0 | 0.009 |
| [KEGG_TIGHT_JUNCTION](http://www.gsea-msigdb.org/gsea/msigdb/cards/KEGG_TIGHT_JUNCTION) | 132 | 0.57 | 2.03 | 0 | 0.007 |
| [KEGG_BASAL_TRANSCRIPTION_FACTORS](http://www.gsea-msigdb.org/gsea/msigdb/cards/KEGG_BASAL_TRANSCRIPTION_FACTORS) | 35 | 0.73 | 2.02 | 0 | 0.008 |
| [KEGG_ENDOCYTOSIS](http://www.gsea-msigdb.org/gsea/msigdb/cards/KEGG_ENDOCYTOSIS) | 180 | 0.6 | 1.99 | 0 | 0.011 |
| [KEGG_EPITHELIAL_CELL_SIGNALING_IN_HELICOBACTER_PYLORI_INFECTION](http://www.gsea-msigdb.org/gsea/msigdb/cards/KEGG_EPITHELIAL_CELL_SIGNALING_IN_HELICOBACTER_PYLORI_INFECTION) | 68 | 0.64 | 1.98 | 0 | 0.01 |
| [KEGG_PURINE_METABOLISM](http://www.gsea-msigdb.org/gsea/msigdb/cards/KEGG_PURINE_METABOLISM) | 156 | 0.58 | 1.98 | 0 | 0.009 |
| [KEGG_AMYOTROPHIC_LATERAL_SCLEROSIS_ALS](http://www.gsea-msigdb.org/gsea/msigdb/cards/KEGG_AMYOTROPHIC_LATERAL_SCLEROSIS_ALS) | 53 | 0.61 | 1.98 | 0 | 0.009 |
| [KEGG_ERBB_SIGNALING_PATHWAY](http://www.gsea-msigdb.org/gsea/msigdb/cards/KEGG_ERBB_SIGNALING_PATHWAY) | 87 | 0.63 | 1.98 | 0.002 | 0.009 |
| [KEGG_RNA_DEGRADATION](http://www.gsea-msigdb.org/gsea/msigdb/cards/KEGG_RNA_DEGRADATION) | 58 | 0.72 | 1.98 | 0 | 0.008 |
| [KEGG_PYRIMIDINE_METABOLISM](http://www.gsea-msigdb.org/gsea/msigdb/cards/KEGG_PYRIMIDINE_METABOLISM) | 98 | 0.65 | 1.97 | 0 | 0.008 |
| [KEGG_TGF_BETA_SIGNALING_PATHWAY](http://www.gsea-msigdb.org/gsea/msigdb/cards/KEGG_TGF_BETA_SIGNALING_PATHWAY) | 85 | 0.6 | 1.97 | 0 | 0.009 |
| [KEGG_MAPK_SIGNALING_PATHWAY](http://www.gsea-msigdb.org/gsea/msigdb/cards/KEGG_MAPK_SIGNALING_PATHWAY) | 267 | 0.54 | 1.96 | 0 | 0.009 |
| [KEGG_ADIPOCYTOKINE_SIGNALING_PATHWAY](http://www.gsea-msigdb.org/gsea/msigdb/cards/KEGG_ADIPOCYTOKINE_SIGNALING_PATHWAY) | 67 | 0.61 | 1.95 | 0 | 0.009 |
| [KEGG_RIG_I_LIKE_RECEPTOR_SIGNALING_PATHWAY](http://www.gsea-msigdb.org/gsea/msigdb/cards/KEGG_RIG_I_LIKE_RECEPTOR_SIGNALING_PATHWAY) | 71 | 0.57 | 1.95 | 0 | 0.009 |
